# Supplementary material for: Advancing COVID-19 diagnostics: rapid detection of intact SARS-CoV-2 using viability RT-PCR assay
Source: Microbiol Spectr. 2024 Jul 22;12(9):e00160-24. doi: 10.1128/spectrum.00160-24 (PMC11370235; doi:10.1128/spectrum.00160-24)
Supplement: Table S1 — Ct values of PMAxx treated and non-PMAxx-treated samples and ΔCt values. [file spectrum.00160-24-s0001.docx]

**SUPPLEMENTARY DATA**

|  | High VL cultured virus | | | Low VL cultured virus | | | Clinical samples | | |
| --- | --- | --- | --- | --- | --- | --- | --- | --- | --- |
| % intact  SARS-CoV-2 | Ct value  -PMAxx | Ct value +PMAxx | ΔCt value | Ct value  -PMAxx | Ct value +PMAxx | ΔCt value | Ct value  -PMAxx | Ct value +PMAxx | ΔCt value |
| 100 | 16.0 | 18.0 | -2.1 | 30.0 | 32.5 | -2.4 | 20.5 | 21.0 | -0.5 |
| 50 | 15.8 | 19.3 | -3.5 | 30.0 | 33.8 | -3.8 | 21.7 | 22.5 | -0.8 |
| 10 | 16.2 | 22.5 | -6.2 | 30.1 | 35.9 | -5.8 | 23.1 | 25.7 | -2.6 |
| 1 | 16.4 | 25.9 | -9.4 | 30.2 | 42.0 | -8.5 | 25.7 | 30.2 | -4.5 |
| 0.1 | 16.2 | 28.5 | -12.3 | 30.4 | UD | ≥11.6 | 24.2 | 31.0 | -6.8 |
| 0 | 16.0 | 30.2 | -14.2 | 30.5 | UD | ≥11.5 | 24.6 | 31.7 | -7.1 |

**Table 1.** Cycle threshold (Ct) values of PMAxx treated and non-PMAxx-treated samples and delta Ct (ΔCt) values for both high and low viral load (VL) cultured virus and clinical samples. ΔCt values are calculated by subtracting the Ct value of the PMAxx-treated sample from the non-PMAxx treated sample. UD, undetectable: no detectable Ct value that exceeded the threshold within 42 cycles.
